# Extracts from *Cephalaria Uralensis* (Murray) Roem. & Schult. and *Cephalaria Gigantea* (Ledeb.) Bobrov as Potential Agents for Treatment of Acne Vulgaris: Chemical Characterization and In Vitro Biological Evaluation

**DOI:** 10.3390/antiox9090796

**Published:** 2020-08-26

**Authors:** Małgorzata Chrząszcz, Małgorzata Miazga-Karska, Katarzyna Klimek, Sebastian Granica, Dorota Tchórzewska, Grażyna Ginalska, Katarzyna Szewczyk

**Affiliations:** 1 Department of Pharmaceutical Botany, Medical University of Lublin, 1 Chodźki Str., 20-093 Lublin, Poland; malgorzata.chrzaszcz329@gmail.com; 2 Department of Biochemistry and Biotechnology, Medical University of Lublin, 1 Chodźki Str., 20-093 Lublin, Poland; malgorzata.miazga-karska@umlub.pl (M.M.-K.); katarzyna.klimek@umlub.pl (K.K.); g.ginalska@umlub.pl (G.G.); 3 Department of Pharmacognosy and Molecular Basis of Phytotherapy, Medical University of Warsaw, 02-097 Warsaw, Poland; sgranica@wum.edu.pl; 4 Department of Plant Anatomy and Cytology, Maria Curie-Skłodowska University, Akademicka 19, 20-033 Lublin, Poland; dorota.tchorzewska@poczta.umcs.lublin.pl

**Keywords:** Cephalaria, LC-DAD-MS^3^, phenolics, antioxidant activity, antiacne activity, cytotoxicity, therapeutic index

## Abstract

The aim of this study was to compare the chemical composition, as well as antioxidant, anti-inflammatory, antiacne, and cytotoxic activites of various extracts of *Cephalaria gigantea* and *C. uralensis*. It is worth underlining that we are the first to characterize the composition and evaluate the biological properties of extracts from *Cephalaria gigantea* and *C. uralensis.* Thus, the LC-DAD-MS^3^ analysis revealed the presence of 41 natural products in studied extracts. The 5-*O*-caffeoylquinic acid, isoorinetin, and swertiajaponin were the main detected compounds. Among the tested samples, ethanol extract of the aerial parts of *C. uralensis* (CUE) possessed the most suitable biological properties. It exhibited moderate ability to scavenge free radicals and good capacity to inhibit cyclooxygenase-1, as well as cyclooxygenase-2. Moreover, CUE possessed moderate antibacterial activity against all tested bacterial strains (*S. aureus*, *S. epidermidis*, and *P. acnes*), and importantly, it was non-toxic towards normal skin fibroblasts. Taking into account the value of calculated therapeutic index (>10), it is worth noting that CUE can be subjected to in vivo study. Thus, CUE constitutes a very promising antiacne agent.

## 1. Introduction

Among all skin diseases that are marked by an abnormal function (usually hyperactivity) of the sebaceous glands in the skin, acne vulgaris is the most common, accounting for 99% of acne cases [1]. The prevalence of acne vulgaris globally was 681.2 million in 2016, which was an increase of 10% from 612 million in 2006. In 2010, acne ranked number 8 in the list of most prevalent diseases in the world, with a global prevalence of 645 million. Approximately 80% of people are affected by acne between the onset of puberty and 30 years of age. In the USA, the cost of acne in terms of treatment and loss of productivity is over 3 billion dollars per year [1].

Acne vulgaris, like other sebborheic disorders, is a chronic inflammatory disorder of the pilosebaceous unit with multifactorial etiology. There are four key processes in its pathogenesis: increased sebum production, follicular hyperkeratinization, which leads to follicular obstruction, colonization by the causative agent, *Cutibacterium acnes* (=*Propionibacterium acnes*), and host inflammatory responses triggered as a result of bacterial infection [2,3]. As a result of the increased sebum production due to high androgen levels, *C. acnes* produces various hydrolytic enzymes that act on the sebum to release free fatty acids. These free fatty acids act as chemokines and increase the release of pro-inflammatory cytokines like interleukins 8 (IL-8) and tumor necrosis factors (TNF-α), which attract macrophages, and lead to severe inflammation. Follicular wall ruptures due to the action of hydrolytic enzymes cause oxidative damage with the release of free radicals [2,3].

Since the causes of seborrheic diseases are multidirectional, the treatment must also be multidirectional. Therapeutic agents should be used to reduce sebum production and reduce follicular hyperkeratinization of the epidermal cells, but it is also necessary to use antibiotic drugs. Such a complex system should be used not only in the treatment of seborrheic diseases—including acne—but also for prevention in people exposed to such skin dysfunctions [4]. Importantly, the treatment of acne and other seborrheic diseases has raised significant clinical challenge due to the increasing appearance of multidrug-resistant pathogens and the high frequency of recurrent lesions [5]. The practice of writing prescriptions for antibiotics to cure seborrheic diseases without recognizing the disease-causing bacterial strain has led to increased resistance among pathogens and decreased efficacy of systemic and topical therapies. Although clinical signs of seborrheic diseases have been ameliorated by numerous antibiotics, bacteria persistence, and increasing resistance to widely used antibiotics are a major problem and underline the need for a new approach to prevent and treat bacterial infections [6]. The global spread of multidrug-resistant bacteria has resulted in the development of the conception of re-establishment of traditional therapeutics [7]. It has been suggested that the current drug discovery approach of finding ‘new entity drugs’, if shifted to ‘combining existing agents’ may be helpful. Therefore, natural product drug discovery based on ethnopharmacology and traditional medicines may also be considered as attractive strategic options [8]. The World Health Organization’s Commission on Intellectual Property and Innovation in Public Health has also recognized the promise and role of traditional medicine in drug development for affordable health solutions [9]. Moreover, the global pharmaceutical industry is looking for innovative solutions to expedite the discovery process. Therefore, innovative approaches inspired by traditional knowledge may accurately occupy this alcove strategy to expedite drug discovery and development process, especially in the existing global economic environment. Traditional medicine certainly offers a sound rationale, valuable experiential wisdom, and a large database of botanical resources. For several reasons, researchers involved in the modern drug discovery have started revisiting ancient traditional knowledge and ethnopharmacology, especially to develop new, effective synergistic drug combinations for management of difficult to treat conditions like seborrheic skin diseases. A paradigm shift in innovation strategy is needed, involving, among other things, revisiting the vast possibilities that the global traditional knowledge bases offer [10]. What is more, the technical developments permitting for detailed chemical characterization together with using modern methods of testing bioactivity led to the re-emergence of natural products for new drug discovery [11].

In the past, medicinal plants were widely used in traditional European medicine for the treatment of seborrheic skin diseases. Plants such as *Prunus africana*, *Glycyrrhiza glabra*, *Melaleuca alternifolia*, *Cephalaria setosa, Cephalaria syriaca,* and many others have been used since ancient times in the treatment of skin diseases [12,13,14,15]. Although the effectiveness of most of these plants in seborrheic skin diseases remains unresolved because of the lack of appropriate scientific evidence. Considering the reported research [12,13,16], we assumed that some groups of compounds included in *Caprifoliaceae* could possess the capacity to inhibit the growth of *P. acnes* and other bacteria that cause skin lesions, scavenge free radicals, and suppress the inflammatory response, which is a very promising approach for the treatment of seborrheic skin diseases.

The overriding idea of our study was to provide comprehensive in vitro study results on the effects of two *Cephalaria* species on seborrheic lesions, with particular emphasis on acne. *Cephalaria* is a genus that includes about 90 species belonging to the Caprifoliaceae family. These annual and perennial plants [17,18] occur mainly in Europe, East Asia, East Mediterranean and Central Africa [19]. *Cephalaria* species are rich in triterpene saponins [20,21,22,23], iridoids [24], alkaloids [25] and flavonoids [26], which are responsible for, for example, cytotoxic [23,27], immunomodulatory [23], antibacterial [17,28], antifungal [29] and hypoglycemic [30] activities. 

## 2. Materials and Methods 

### 2.1. Chemicals and Reagents 

LC-grade acetonitrile, ascorbic acid, 2,2-diphenyl-1-picrylhydrazyl radical (DPPH^•^), 2,20-azino-bis-(3-ethyl-benzothiazole-6-sulfonic acid) (ABTS^●+^), indomethacin and disodium dihydrate (Na_2_EDTA*2H_2_O), were obtained from Sigma-Aldrich Fine Chemicals (St. Louis, MO, USA). Phosphate-buffered saline (PBS) was obtained from Gibco (Carlsbad, CA, USA). Reference compounds were purchased from ChromaDex (Irvine, CA, USA). Water and formic acid for LC analysis were from Merck (Darmstadt, Germany). All the other chemicals were of analytical grade and were purchased from the Polish Chemical Reagent Company (POCH, Gliwice, Poland).

### 2.2. Plant Material 

The aerial parts and flowers of *Cephalaria uralensis* (Murray) Roem. & Schult. and the aerial parts of *C. gigantea* (Ledeb.) Bobrov were collected in the Maria Curie-Skłodowska University (UMCS) Botanical Garden in Lublin (Poland), at an altitude of 181 m a.m.s.l. coordinates N 51°15′40” E 22°30′51” in August 2019. Taxonomical identification was confirmed by Dr A. Dąbrowska, the botanist from the Botanical Garden in Lublin. 

### 2.3. Preparation of the Extracts

The plant materials were dried in shade and at 26 °C (±0.5 °C) until constant weight [31]. Extracts were prepared using 80% acetone (3 × 300 mL), mixture of methanol-acetone-water (3:1:1, *v/v/v*; 3 × 300 mL) and 30% ethanol (1 × 300 mL) [32]. With acetone and mixture of methanol-acetone-water, plant materials were sonicated at controlled temperature (40 ± 2 °C) for 30 min, and with ethanol were shaken at room temperature for 24 h. The combined extracts were filtered, concentrated under reduced pressure, and after freezing, lyophilized in a vacuum concentrator (Free Zone 1 apparatus; Labconco, Kansas City, KS, USA) to obtain dried residues. 

### 2.4. LC-ESI-MS/MS Analysis 

The LC analysis was performed using Ultimate 3000 series system (Dionex, Idstein, Germany) equipped with dual low-pressure gradient pump with vacuum degasser, an autosampler, a column compartment, and a diode array detector coupled with Amazon SL ion trap mass spectrometer (Bruker Daltonik GmbH, Bremen, Germany). The separation of compounds in the analyzed extract was carried out with Kinetex XB-C_18_ analytical column (100 mm × 2.1 mm × 1.9 µm), Phenomenex (Torrance, CA, USA). Column temperature was maintained at 25 °C. Elution was conducted using mobile phase A (0.1% HCOOH in deionized water) and mobile phase B (0.1% HCOOH in acetonitrile) with a two-step gradient as follows: 0 min 1% B, 60 min 26% B and finally 90 min 95% B. The flow rate was set to 0.300 mL/min. Five µL of each sample was introduced to the column by the autosampler. UV-vis spectra were recorded in the range of 190–450 nm. Chromatograms were acquired at 254 nm. The eluate was introduced directly into mass spectrometer. The ion trap mass spectrometer was equipped with ESI interface. The parameters for ESI source were set as follows: nebulizer pressure 40 psi; dry gas flow 9 L/min; dry temperature 300 °C; and capillary voltage 4.5 kV. Analysis was carried out using a scan from *m*/*z* 70–2,200. Compounds were analyzed in negative ion mode. The MS^2^ fragmentations were performed using Smart Frag mode.

#### Quantification of Major Compounds by UHPLC–DAD

The quantification of the major compounds was performed using the analytical method reported in Section 2.4 and based on the calibration curves of various compounds: luteolin for the quantification of flavonoid derivatives, aucubin for iridoids, chlorogenic acid for phenolic acids and unidentified compounds. The areas of quantified compounds were determined at 275, 280 and 350 nm. For the quantification, accurately weighed 10 mg of DE was dissolved in 1 mL of methanol/water mixture (1:1, *v/v*). Samples were analyzed in triplicate. Stock solutions of the standards were prepared by dissolving 1 mg of the compounds in 1 mL of methanol. The stock solutions were diluted in mobile phase to obtain solution at the concentration of 50 µg/mL. Different amounts of the standard were injected into the UHPLC column. The calibration curves were plotted based on the amount of injected compounds vs. peak area at 280 and 350 nm. The linear range was 50–400 ng/injection (R^2^ ≥ 0.99).

### 2.5. Antioxidant Activity 

All assays were performed using 96-well microplates (Nunclon, Nunc, Roskilde, Denmark) and were measured in an Infinite Pro 200F Elisa Reader (Tecan Group Ltd., Männedorf, Switzerland). Results were expressed as the IC_50_ values of the *Cephalaria* extracts based on concentration–inhibition curves.

#### 2.5.1. DPPH^●^ Assay

2,2-diphenyl-1-picryl-hydrazyl (DPPH^●^) free radical scavenging activity of *Cephalaria* extracts and the reference ascorbic acid was tested using a modified method, described previously [32]. Decreasing of DPPH^●^ absorbance, induced by the extracts was monitored at 517 nm after incubation at 28 °C for 30 min. Ascorbic acid was used as a positive control.

#### 2.5.2. ABTS^●+^ Assay

The second method used was 2,2′-azinobis[3-ethylbenzthiazoline]-6-sulfonic acid (ABTS^●+^) decolorization assay [32]. The absorbance was measured at 734 nm. Trolox was used as a positive control.

#### 2.5.3. Metal Chelating Activity

The metal chelating activity was determined using the method described by Guo et al. [33], modified in our previous study [32]. The absorbance was measured at 562 nm. Na_2_EDTA*2H_2_O was used as a positive control.

### 2.6. Anti-Inflammatory Activity

The extracts of *Cephalaria* species were tested for cyclooxygenase-1 (COX-1) and cyclooxygenase-2 (COX-2) inhibitory activity using a COX (ovine/human) Inhibitor Screening Assay Kit (Cayman Chemical, MI, USA) according to the protocol of the manufacturer. The stock solution of the studied extracts were dissolved in ethanol, and their final concentration was 50 and 100 µg/mL. Indomethacin was used as a positive control for inhibition of COX-1 and COX-2.

### 2.7. Antimicrobial Activity

#### 2.7.1. Bacterial Strains

*Cephalaria gigantea* and *C. uralensis* extracts were screened for their in vitro antibacterial activity against acne strains: Gram-positive aerobic *Staphylococcus aureus* ATCC 25923, *Staphylococcus epidermidis* ATCC 12228 and microaerobic *Propionibacterium acnes* PCM 2400, *Propionibacterium acnes* PCM 2334. For antibacterial activity determination, Mueller-Hinton agar or broth (MH-agar, MH-broth) for aerobic and Brain-Heart Infusion agar or broth (BHI-agar, BHI-broth) for microaerobic strains were used. Bacterial inoculums were prepared by subculturing microorganisms into MH-agar or BHI-agar at 37 °C for 24 or 48 h, respectively. The growth was harvested using 5 mL of 0.9% NaCl and diluted to 0.5 McFarland (1.5 × 10^8^ CFU/mL (CFU: colony-forming unit)).

#### 2.7.2. Agar Disc Diffusion Assay

The antibacterial activity of all samples was initially performed by a modified disc diffusion method based on Murray recommendations [34]. The bacterial inoculum was spread on the surface of the Petri plates containing agar, using a cotton swab. The solutions of all tested extracts (100 µg/mL) were placed on inoculated Petri plates. Plates with MH-agar (for aerobic strains) were incubated for 24 h at 37 °C and plates with BHI-agar for 48 h at 37 °C. The diameter of the growth inhibition zone around each compound was measured after incubation.

#### 2.7.3. MIC and MBC Determination

The minimum inhibitory concentration (MIC) of plants extracts was determined for bacterial strains, which exhibited the bacterial growth inhibition zones. The test was performed using double serial microdilution in the 96-well microtiter plates according to CLSI method with some modifications [35]. The 200 µL of broth was pipetted into each well. The double serial dilution of tested derivatives was performed in the test wells causing rise to concentrations ranging from 200 µg/mL to 1.562 µg/mL. Finally, 2 µL of tested bacteria inoculum was added to the wells (except negative sterility control). The tests were performed either at 37 °C for 24 h (aerobic strains) or 48 h (microaerobic strains). After incubation, the panel was digitally analyzed at 600 nm using the microplate reader Bio-Tech Synergy (USA) with a dedicated software system. The growth intensity in each well was compared with the negative and positive controls. Additionally, minimal bactericidal concentration (MBC) was determined by spreading on agar medium (10 µL) from a clear well, which did not show any visible growth after incubation after MIC test. The plates were incubated at 37 °C for 24 h, and the MBC was defined as the lowest concentration of sample without bacterial growth. Each experiment was repeated in triplicate.

#### 2.7.4. Synergy Test

Synergistic interactions between CUM, CUE, CUA extracts and antibiotics: Ceftriaxone (Polpharma S.A., Starogard Gdański, Poland), Cefepime (Bristol-Myers Squibb, Sermoneta, Italy), Sparfloxacin (Dainippon Pharmaceutical CO., LTD, Cheshire, UK), and Ciprofloxacin (Polpharma S.A., Starogard Gdański, Poland) were examined by the checkerboard test based on MIC determination. The stock solutions and serial two-fold dilutions of antibiotics and extracts CUM, CUE, CUA to fourfold the MIC were prepared. A total of 50 μL of Mueller-Hinton broth was distributed into each well of the microdilution plates. The first antibiotic of the combination was serially diluted vertically, while the plant extract was diluted horizontally in the 96-wells plate. Next, 100 μL of bacterial inoculum of 1.5 × 10^8^ CFU/mL, was added and the plates were incubated in appropriate conditions. The resulting checkerboard contained each combination of two tested agents. The plate layout also included the MIC determination of agents used separately. The fractional inhibitory concentration index (FICI) was calculated for each combination of two agent’s concentrations according to the following formula:

FICI = (MIC_A/B_/MIC_A_) + (MIC_B/A_/MIC_B_) (1)

where MIC_A_ = MIC of the agent A alone, MIC_A/B_ is the MIC of agent A in combination with agent B and MIC_B_, MIC_B/A_ is defined analogously as agent A.

The obtained values of the FICI indicated: total synergism (FICI ≤ 0.5), partial synergism (0.5 < FICI ≤ 0.75), no effect (0.75 < FICI ≤ 2) or antagonism (FICI > 2) between two agents [36].

### 2.8. Cytotoxicity Evaluation

Cytotoxicity assessment was carried out using normal human skin fibroblasts (BJ cell line, ATCC CRL-2522^™^), as described in detail previously [37]. Before the test, the extracts possessing the best antibacterial activity (zones of bacterial growth inhibition above 10 mm) were selected and their stock solutions were prepared at a concentration of 100 mg/mL in dimethyl sulfoxide (DMSO). Then, two-fold serial dilutions of extracts (ranging from 1000–1.95 μg/mL) in culture medium were obtained and the received solutions were incubated with BJ cells for 24 h at 37 °C in a humidified atmosphere (5% CO_2_, 95% air). The cell viability was evaluated via MTT assay according to the procedure described previously [38]. The results (*n* = 3) were expressed as mean values ± standard deviation (SD). Moreover, based on obtained results, the values of half-maximum cytotoxic concentration (CC_50_) were calculated via four-parameter nonlinear regression analyses (GraphPad Prism 5, version 5.04). The CC_50_ means a concentration of extract required for reduction of BJ cell viability to 50%. To determine the potential safety of each extract, therapeutic index (TI) was calculated. The TI indicates the value, which is calculated as a ratio of CC_50_ (cytotoxic activity) and MIC (antibacterial property). Higher TI values indicate that the extract exhibits greater safety towards eukaryotic cells.

### 2.9. Statistical Analysis

The results were expressed as mean values ± standard deviation (SD) of the indicated number of experiments. The IC_50_ values of investigated extracts were calculated, based on concentration–inhibition curves. Statistical significance of differences between means was established by one-way ANOVA with Dunnett’s post hoc test. *p*-Values below 0.05 were considered statistically significant. The data from cell culture experiments were subjected to statistical analysis using unpaired Student’s *t*-test and differences were considered significant when *p* < 0.05 (GraphPad Prism 5, version 5.04, San Diego, California, U.S.).

## 3. Results and Discussion

### 3.1. Qualitative LC-DAD-MS^3^ Analysis of the Chemical Composition of C. uralensis and C. gigantea

**Figure 1:**
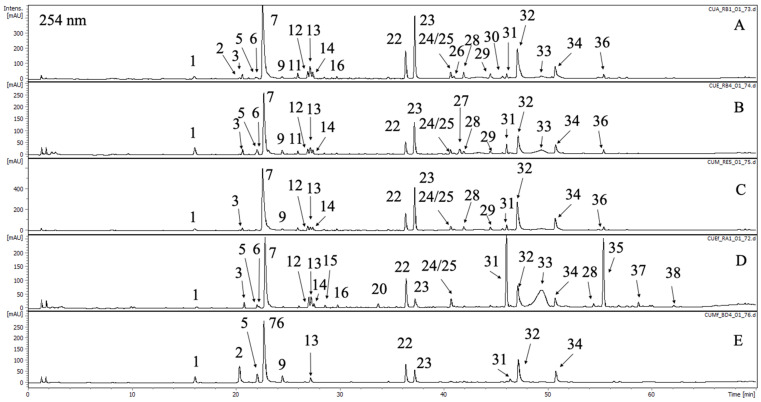
The LC-DAD chromatogram of *C. uralensis* acquired at 254 nm with marked compounds. Peak numbers refer to those implemented in Table 1. A—CUA, B—CUE, C—CUM, D—CUEf, E—CUMf.

**Figure 2:**
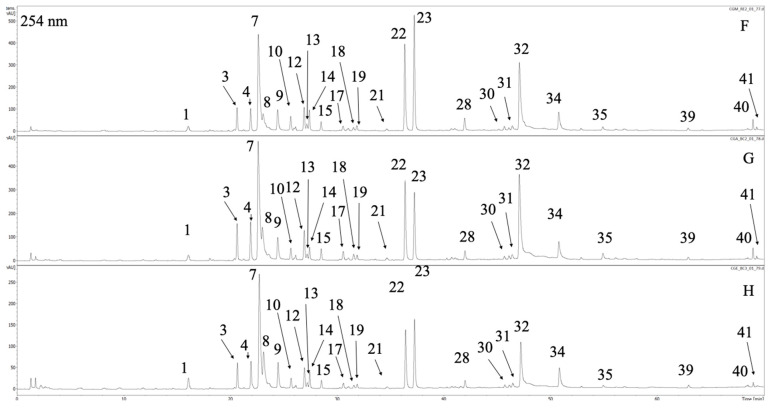
The LC-DAD chromatogram of *C. gigantea* acquired at 254 nm with marked compounds. Peak numbers refer to those implemented in Table 1. F—CGA, G—CGE, H—CGM.

The phytochemical analysis of extracts of *C. uralensis* and *C. gigantea* revealed the presence of forty-one constituents, which were characterized based on UV-Vis spectrum and MS/MS spectra. Compounds with intensive UV-Vis maxima at ca. 325 nm, namely **1**, **2**, **5**, **6**, **7**, **9**, **11**, **13**, **25**, **27**, **30**, **32**, and **34**, were classified as caffeic acid derivatives. Compounds **1**, **6**, **9**, and **11** showed deprotonated ions in the mass spectrum at *m*/*z* = 353. According to the comparison with literature and chemical standards available **1**, **6**, and **9** were identified as neochlororgenic acid, chlorogenic acid and cryptochlorogenic acid, respectively [39]. Peaks **6** and **11** had similar mass spectra to the latter three and they were assigned as caffeoylquinic acid isomers, most probably *cis* chlorogenic acids [40]. Two dicaffeoylquinic acids showing base peak ions in MS spectrum as *m*/*z* = 515 were detected and labelled as **32** and **34**. According to Clifford et al., they were identified as 3,5- and 4,5-dicaffeoylquinic acids [41]. Compounds **5** and **27** showed deprotonated peaks at *m*/*z* = 179 and 193, respectively. Based on previous reports on chemical composition of *Cephalaria* species and after the comparison with standards they were assigned as caffeic and ferulic acids [42]. Compounds **2**, **25** and **30**, with base peak signals in the mass spectra at *m*/*z* = 369, 545 and 587, were assigned as unspecified phenolic acid derivatives. The second subgroup of phenolics abundant in the analyzed extracts were flavonoids and their derivatives (Table 1, Figure 1 and Figure 2). They showed specific maxima in UV-Vis spectra at ca. 335 for apigenin derivatives (**21**, **24**, **28**), and ca. 350 for luteolin and quercetin glycosides (**22**, **23**, **26**). Compound **26** showed deprotonated ion at *m*/*z* = 463. The fragmentation revealed the cleavage of hexose unit (M-162) and the presence of aglycone residue at *m*/*z* = 301. The comparison with the chemical standard proved that **26** is quercetin 3-*O*-galactoside (hyperoside). This compound was confirmed in *Cephalaria grossheumii* Bobr. species [26]. Compounds **21**, **24** and **28** were identified as apigenin derivatives. Compound **28** showed base peak ion at *m*/*z* = 431 and characteristic fragmentation pattern typical for *C*-glycosides. Based on previous reports compound **28** was assigned as isovitexin [43]. Compound **21**, with the major peak ion at *m*/*z* = 593, showed cleavage of hexose unit in the fragmentation spectrum (M-162), resulting in the production of isovitexin moiety at *m*/*z* = 431. Thus, **21** was characterized as isovitexin *O*-hexoside. The latter two flavonoids have not been previously reported from *Cephalaria* species. Compounds **22** and **23** were characterized as luteolin glycosides. Compound **22** showed major ion at *m*/*z* = 447, and the fragmentation of this ion did not lead to the production of aglycone moiety. Instead pattern typical for C-glycosides was observed, thus 22 was assigned as isoorientin [43]. Compound **23** showed characteristic fragmentation pattern for swertiajaponin and has been previously reported from other *Cephalaria* species [44]. Compounds **31**, **38**–**40**, due to the fact of molecular weights in the rage of 580-640 amu and intensive maximum in the UV-Vis spectra at ca. 260 and 315 nm, were classified as flavonoid with phenolic acid moiety linked to sugar residue. After comparison with the chemical standard compounds, **30** was identified as tiliroside, which has been previously detected in *Cephalaria elmanensis* [45]. Compounds **31**, **40** and **41** may be assigned as kaempferol glycosides with *p*-coumaroyl moiety, because of the fact that their fragmentation showed cleavage of 146 amu resulting in the production of fragment ions M-146 at *m*/*z* = 601, 489 and 489, respectively (Table 1).

The last class of natural products occurring in analysed extract were iridoids (**3**, **4**, **8**, **12**, **14**, **15**–**20**, and **29**). Compounds **3** and **4** showed deprotonated ion as *m*/*z* = 375 with UV-Vis maximum at 231 nm. Based on comparison with chemical standard compound **3** was identified as loganic acid, which was previously confirmed in *Cephalaria kotshyi* [46]. Compound **4** was described as loganic acid isomer due to the same molecular weight and identical fragmentation pattern (Table 1). Compound **12** showed deprotonated ion in the mass spectrum at *m*/*z* = 359. The fragmentation pattern was like compounds **3** and **4**. Based on literature review, compound **12** was tentatively identified as deoxyloganic acid [47]. Compound **15** had major ion in the mass spectrum at *m*/*z* = 435 corresponding to M+HCOO^●−^ adduct (Table 1). After the comparison with chemical standard, **15** was identified as loganin. Loganin was previously confirmed in *Cephalaria elaziginesis*, *Cephalaria kotschyi* and *Cephalaria isaurica* [27,46,48]. Compounds **8**, **16**–**20** and **29** were tentatively classified as other iridoids based on UV-Vis maxima observed at ca. 240 nm and mass spectra identified compounds.

The quantitative evaluation of the major compounds detected in the analyzed extracts was performed based on the calibration curves of different compounds: luteolin for the quantification of flavonoid derivatives, aucubin for iridoids, chlorogenic acid for phenolic acids and unidentified compounds. The areas of quantified components were determined at 275, 280 and 350 nm. Among the analyzed compounds, 5-*O*-caffeoylquinic acid, 3,5-*O*-dicaffeoylquinic acid, isoorientin and swertiajaponin were presented in all samples in the greatest quantities. It was found that the most effective solvent in extracting phenolics in both both *Cephalaria* species was 80% acetone followed by mixture of methanol–acetone–water; this may be due to the lower water content and it is in agreement with the other reports [49]. The leading phenolic acids identified in both species were 5-*O*-caffeoylquinic acid (94.90 ± 0.42–135.83 ± 1.47 µg/g DE) and 3,5-*O*-dicaffeoylquinic acid (41.29 ± 0.11–118.90 ± 0.46 µg/g DE). Among flavonoids the most abundant compounds were isoorientin (41.71 ± 0.50–108.42 ± 0.79 µg/g DE) and swertiajaponin (7.91 ± 0.08–115.10 ± 0.45 µg/g DE). In both *Cephalaria* species there are more caffeic acid derivatives in contrast to the iridoids and flavonoids content. These compounds are important ingredients in dermatological products through reduction of inflammation, oxidative stress and inhibition the growth of bacteria, such as *P. acnes* and *S. aureus* [50].

### 3.2. Antioxidant Activity

Based on the hypothesis that oxygen free radicals are involved in the pathogenesis of acne because of their activities on neutrophils [51], we evaluated the free radical scavenging effects of *Cephalaria* extracts using DPPH^●^ and ABTS^●+^ assays. The measurement of antioxidant activities was performed on a microplate scale in cell-free systems. All extracts were studied in the concentration range from 0.16 to 10 mg/mL and they exhibited similar moderate scavenging ability in a concentration-dependent manner. In comparison, the radical scavenging activity of ascorbic acid was measured in the same conditions. The higher DPPH^●^ scavenging activity was demonstrated for the CUM extract (IC_50_ = 2.86 ± 0.12 mg/mL) followed by CUA (IC_50_ = 4.42 ± 0.53 mg/mL), CGE (IC_50_ = 4.90 ± 0.62 mg/mL), and CUE (IC_50_ = 5.55 ± 0.10 mg/mL). However, the IC_50_ values for *Cephalaria* extracts were higher than this for ascorbic acid (IC_50_=0.48 ± 0.30 mg/mL) (Table 2).

Slightly different results were observed in ABTS^●+^ assay, where CUMf extract was found to scavenge free radical strongly (IC_50_ = 0.45 ± 0.21 mg/mL) followed by CGA (IC_50_ = 0.53 ± 0.16 mg/mL), and CGM (IC_50_ = 0.54 ± 0.21 mg/mL).

The chelating capacity was based on measuring the percentage of inhibition of ferrozine-Fe^2+^ complex formation. The CUM and CUA extracts were the most active ones interfering with the formation of iron and ferrozine complexes, which suggest their high chelating capacity and ability to capture iron ions before ferrozine. These extracts had IC_50_ values comparable (0.25 ± 0.11 mg/mL and 0.49 ± 0.17 mg/mL, respectively) to Na_2_ EDTA*2H_2_O (IC_50_ = 0.01 mg/mL), which was used as positive control.

The results of various reports can be difficult to compare due to the different conditions of the experiments used. Moreover, the state of knowledge of the antioxidant activity of *Cephalaria* species is fragmentary. Azab [52] found that aqueous, ethanolic and ethyl acetate extracts of the aerial parts of *C. jopponsis* possess high antioxidant activity (41.1, 30.1 and 20.7 mg of ascorbic acid g^−1^ of dry extract, respectively).

The main compounds identified in most active extracts (CUM and CUA) are chlorogenic acid, isoorientin and swertiajaponin. These compounds are well-known natural antioxidants showing strong effects in different tests [53].

Sarici and co-authors [51] found that in acne vulgaris, the antioxidant defense system is damaged; thereby, it may be concluded that plants with high antioxidant activity such as *Cephalaria* species can be indicated for the treatment of acne.

### 3.3. Anti-Inflammatory Activity

It is well known that *Propionibacterium acnes* release pro-inflammatory enzymes and chemotactic factors, and that inflammation is an integral factor in the pathophysiology of acne [54].

In our study, COX-1 and COX-2 enzymes inhibitory activities of extracts of *C. uralensis* and *C. gigantea* were investigated as a mechanism of their anti-inflammatory action. COX catalyzes the conversion of arachidonic acid into prostaglandins, which play a significant role in health and several diseases. It exists in two isoforms—constitutive COX-1 (responsible for maintaining normal physiological function) and inducible COX-2 (its expression is activated in inflammatory conditions). The inhibition of COX-1 causes some side-effects, while the inhibition of COX-2 provides therapeutic effects in inflammation, pain, and many other diseases [55].

In the present study, for the first time to our knowledge, the anti-inflammatory activity of *Cephalaria* extracts was investigated. The extracts were studied at two concentrations: 50 and 100 µg/mL. The results of the inhibition of both cyclooxygenases were recorded as percentage inhibition of prostaglandin biosynthesis, and these are shown in Table 3. A minimum inhibition of 50% is needed for plant extracts to be considered active [56].

All *C. uralensis* extracts showed good activity against COX-1 and COX-2. The most active against COX-1 was CUM (97.90%) in the concentration of 100 µg/mL, followed by CUMf (94.64%) and CUE (92.54%), while the weakest were extracts of *C. gigantea*—CGM (17.01%) and CGA (32.23%). Except for CUMf and CGM, all extracts at both concentrations studied showed good activity against COX-2 (52.28–71.52% at 50 µg/mL and 55.75–94.85% at 100 µg/mL).

### 3.4. Antimicrobial Activity

All samples were preliminarily tested for their antibacterial activity by a modified disc diffusion method determining zones of bacterial growth inhibition. The size of the bacterial growth inhibition zone is directly proportional to the degree of sensitivity of bacteria against the tested drug. The larger the inhibition zones are, the higher the sensitivity exhibited by the bacteria towards the tested agents. All tested plant extracts showed some antibacterial activity against acne strains. Thus, CUA, CUE, CUMf, CUEf, CGA, CGM, CGE extracts had moderate activity against the tested bacteria, while CUM was considered to be the most active extract (Table 4). Importantly, this extract showed higher activity in agar tests against acne microaerobic strains (21.5–23 mm) than against aerobic bacteria (17–19 mm).

Moreover, the minimum inhibitory concentration (MIC) of compounds was determined for active extracts that exhibited bacterial growth inhibition zones. The MIC values analysis (Table 5) confirmed that CUM extract possessed beneficial inhibitory effect against tested strains, especially towards microaerobic Gram-positive strains *P. acnes 2334* and *P. acnes 2400* with MICs of 12.5 µg/mL. Nevertheless, CUE, CUMf, and CUA extracts also achieved favorable MIC values for bacterial strains in range of 25–100 μg/mL. The strongest antibacterial effect was found in the extracts that have higher swertiajaponin, isoorientin and chlorogenic acids content. This is in accordance with previous research suggesting that these compounds possess strong antimicrobial properties [57].

Additionally, to determine the inhibitory and killing abilities of the tested extracts, the MBC was determined. To consider the *Cephalaria* extracts to have bactericidal activity, the MBC/MIC ratio must be equal or less than 4. The calculated value obtained for CUM was 4, against both *Propionibacterium* strains, what proved its bactericidal properties. The other tested extracts showed MBC values higher than 4; therefore, they have bacteriostatic—not bactericidal—properties.

The antibacterial active extracts (CUM, CUE, CUA) were examined for theoretical synergism with selected antibiotics. The data shown in Table 6 indicate that none of the tested samples of CUM, CUA, CUE had an antagonistic effect when combined with the selected drugs. Importantly, the CUM extract showed a beneficial synergistic effect in combination with ceftriaxone and cefepime on both *Propionibacterium* strains (FICI range 0.375–0.5). Other combinations of drugs and extracts against the tested bacterial strains had no effect.

Some other *Cephalaria* species have already been reported with antibacterial activity. Kırmızıgül and co-authors [22] showed that methanol extract of *C. transsylvanica* possesses antimicrobial activity, i.a., against *S. aureus*, *E. coli*, *P. vulgaris*, *P. aeruginosa*, *C. xerosis,* and *K. pneumoniae*. The antibacterial activity of the *C. elmaliensis* and *C. scoparia* extracts and the pure triterpenoids were also investigated [17,28]. Sarıkahya and Kırmızıgül [17,28] found that compounds, including hederagenin-type triterpene saponins, tiliroside and luteolin 7-*O*-*β*-D-glycoside, isolated from *Cephalaria* species were very active against both gram-positive and gram-negative bacteria.

### 3.5. Cytotoxic Activity

**Figure 3:**
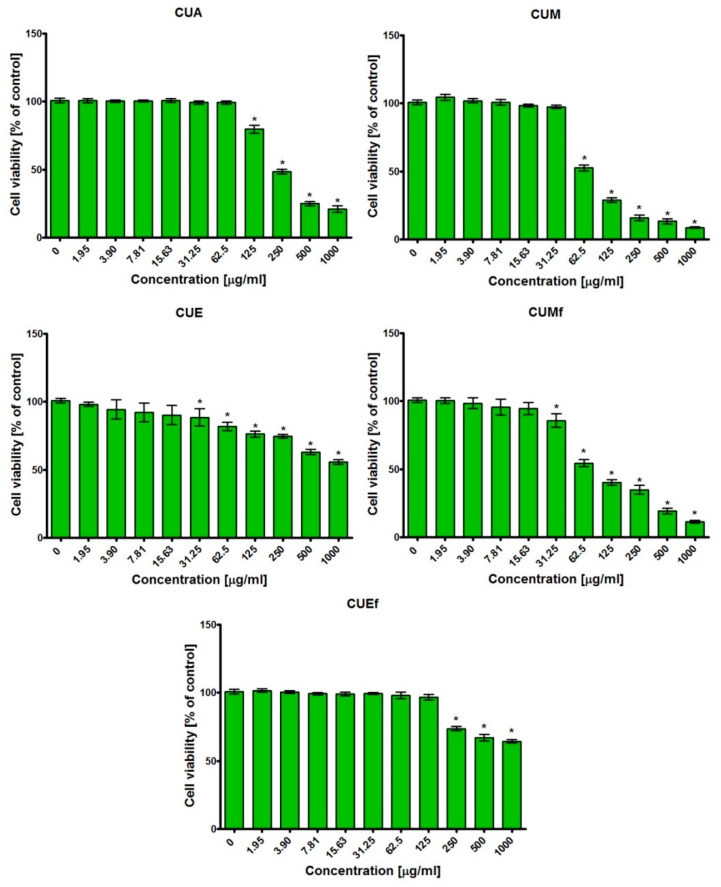
Viability of normal human skin fibroblasts (BJ cell line, ATTC CRL-2522^™^) after 24 h incubation with the most active extracts (CUA, CUM, CUE, CUMf, CUEf). * Significantly different results compared to control (culture medium without extracts—0 μg/mL); unpaired *t*-test, *p* < 0.05.

After 24 h incubation, the MTT test revealed that the tested extracts possessed different abilities to reduce the viability of normal human skin fibroblasts (Figure 3). Thus, at the highest tested concentration (1000 μg/mL) of CUA, CUM, CUE, CUMf, CUEf extracts, the BJ cell viability (compared to control) was 21.06 ± 2.35 μg/mL (*p* < 0.0001), 8.71 ± 0.54 μg/mL (*p* < 0.0001), 55.87 ± 1.72 μg/mL (*p* < 0.0001), 11.47 ± 1.01 μg/mL (*p* < 0.0001), and 64.49 ± 1.22 μg/mL (*p* < 0.0001), respectively. It is worth noting that CUM, which possessed the best antibacterial activity (Table 4 and Table 5) also exhibited the highest cytotoxic activity towards BJ cells, with CC_50_ value equal to 61.27 ± 2.74 μg/mL (Table 7). In turn, CUE and CUEf extracts were found to be non-toxic, because their values of CC_50_ were above 1000 μg/mL. Lack of cytotoxicity and moderate antibacterial activity of these extracts meant that they possessed the highest values of therapeutic indexes (Table 7). Importantly, CUE extract exhibited TI values above 10 against all tested bacterial strains. TI values above 10 indicate that the compound possesses therapeutic potential in vitro and it may be subjected to in vivo evaluation [38]. Therefore, in vitro results indicate that CUE extract seems to be a very promising antibacterial agent. Nevertheless, to confirm its therapeutic potential precisely, the appropriate in vivo study will be performed. For this purpose, an in vivo skin irritation study will be performed on healthy volunteers (without dermatological diseases or allergy to substances occurring in extracts) as described previously [58], The visual assessment will be carried out according to the Hibry and Murdan guidelines [59].

## 4. Conclusions

The management of acne vulgaris should be “multidirectional”. Therapeutic agents should reduce sebum production as well as follicular hyperkeratinization of the epidermal cells. For this reason, they should primarily exhibit antioxidant, anti-inflammatory, and antimicrobial properties, without cytotoxic effect.

In our in vitro study, we characterized composition and comprehensively evaluated biological properties of extracts from *Cephalaria uralensis* and *C. gigantea.* Thus, we identified the main compounds presenting in extracts as well as we determined antioxidant, anti-inflammatory, antimicrobial, and cytotoxic properties of such extracts.

We proved that both species are rich in chlorogenic acid, swertiajaponin, and isoorientin, which are most likely responsible for their beneficial biological activities. Among the tested samples, the ethanol extract of the aerial parts of *C. uralensis* (CUE) possessed the most desirable biological properties, as it had antibacterial, anti-inflammatory, and antioxidant abilities, while also not exhibiting cytotoxic effects towards normal human skin fibroblasts. It is worth underlining that such properties revealed in vitro make it possible to suppose the potential “multidirectional” activity of CUE. Thus, CUE seems to be a promising agent for treatment of acne vulgaris, and for this reason, it will be subjected to further in vivo study in order to confirm its “multidirectional” activity.

## Figures and Tables

**Table 1:** LC-DAD-MS/MS data for compounds detected in the *C. uralensis* and *C. gigantea* extracts. CUA—acetone extract of the aerial parts of *C. uralensis*, CUE—ethanol extract of the aerial parts of *C. uralensis*, CUM—methanol–acetone–water extract of the aerial parts of *C. uralensis*, CUEf—ethanol extract of the flowers of *C. uralensis*, CUMf—methanol-acetone-water extract of the flowers of *C. uralensis*, CGA—acetone extract of the aerial parts of *C. gigantea*, CGE—ethanol extract of the aerial parts of *C. gigantea*, CGM—methanol-acetone-water extract of the aerial parts of *C. gigantea*.

No	Compound Name	Retention Time [min]	UV Maxima [nm]	[M-H]^-^ *m*/*z*	MS^2^ Ions	CUA	CUE	CUM	CUEf	CUMf	CGA	CGE	CGM
1	3-*O*-caffeoylquinic acid (neochlorogenic acid) ^c^	16.1	300sh,324	353	191b, 179	4.43 ± 0.13	8.75 ± 0.62	3.87 ± 0.08	3.42 ± 0.11	8.54 ± 0.42	5.45 ± 0.32	9.35 ± 0.17	5.13 ± 0.18
2	phenolic acid derivative	20.4	300sh 324	369	207b	0.61 ± 0.04	-	-	-	20.65 ± 0.17	-	-	-
3	loganic acid ^s^	20.6	231	375	213b, 169	3.92 ± 0.11	4.60 ± 0.07	0.83 ± 0.02	1.35 ± 0.23	-	13.47 ± 0.23	22.17 ± 0.45	14.05 ± 0.17
4	loganic acid isomer	21.9	231	375	213b, 169	-	-	-	-	-	14.14 ± 0.09	22.45 ± 0.11	14.78 ± 0.25
5	caffeic acid ^s^	22.2	300sh, 325	179	-	0.84 ± 0.19	1.27 ± 0.09	-	0.91 ± 0.05	0.79 ± 0.11	-	-	-
6	caffeoylquinic acid isomer	22.3	300sh, 325	353	191b	4.27 ± 0.10	4.99 ± 0.05	-	1.96 ± 0.02	-	-	-	-
7	5-*O*-caffeoylquinic acid (chlorogenic acid) ^c^	22.8	300sh, 325	353	191b,179	123.75 ± 0.54	114.90 ± 1.06	132.18 ± 1.07	94.90 ± 0.42	98.75 ± 0.61	101.79 ± 0.51	135.83 ± 1.47	130.88 ± 0.77
8	undefined iridoid	23.2	245	389	-	-	-	-	-	-	16.03 ± 0.19	31.48 ± 0.62	22.02 ± 0.15
9	4-*O*-caffeoylquinic acid (cryptochlorogenic acid) ^c^	24.3	299sh, 324	353	191, 179, 173b	1.89 ± 0.01	4.01 ± 0.05	1.68 ± 0.06	-	7.45 ± 0.19	16.02 ± 0.06	20.80 ± 0.16	16.38 ± 0.08
10	undefined compound	27.8	213, 280	355	225, 179b	-	-	-	-	-	14.72 ± 0.09	17.71 ± 0.12	4.97 ± 0.10
11	caffeoylquinic acid isomer	26.1	299, 325	353	191b	3.69 ± 0.05	3.92 ± 0.09	-	-	-	-	-	-
12	deoxyloganic acid ^t^	27.0	242	359	322b, 209	2.14 ± 0.17	2.67 ± 0.42	2.39 ± 0.08	1.85 ± 0.12	-	14.82 ± 0.12	17.05 ± 0.31	15.21 ± 0.62
13	caffeoylquinic acid isomer	27.3	300sh, 324	353	191b	7.50 ± 0.08	4.15 ± 0.01	1.97 ± 0.05	12.62 ± 0.35	2.76 ± 0.02	3.84 ± 0.05	4.85 ± 0.15	3.92 ± 0.01
14	flavonoid derivative	27.5	261, 353	625	607, 535, 505b, 463, 343	8.28 ± 0.18	4.62 ± 0.05	3.51 ± 0.10	2.69 ± 0.07	-	14.89 ± 0.15	8.00 ± 0.09	15.23 ± 0.08
15	loganin ^s^	28.7	249	435f	389, 227b	-	-	-	0.51 ± 0.02	-	4.33 ± 0.12	6.64 ± 0.18	4.56 ± 0.05
16	undefined iridoid	29.9	248	535f	489b, 399, 293, 195	0.17 ± 0.03	-	-	0.47 ± 0.01	-	-	-	-
17	undefined iridoid	30.7	230	433f	387b, 355, 265, 225, 155	-	-	-	-	-	3.41 ± 0.12	5.55 ± 0.21	3.18 ± 0.02
18	undefined iridoid	31.6	230	433f	387b, 355, 265, 225, 155	-	-	-	-	-	2.75 ± 0.11	2.53 ± 0.08	1.61 ± 0.01
19	undefined iridoid	32.0	224	403f	371b, 333, 223, 121	-	-	-	-	-	3.52 ± 0.05	2.72 ± 0.10	1.72 ± 0.01
20	undefined iridoid	33.9	248	488	471, 350, 341b, 281, 146	-	-	-	1.20 ± 0.07	-	-	-	-
21	isovitexin *O*-hexoside	34.8	267, 334	593	473, 431b, 341, 311, 283	-	-	-	-	-	2.82 ± 0.06	2.98 ± 0.05	2.15 ± 0.02
22	isoorientin ^s^	36.5	270, 349	447	429, 357, 327b	65.18 ± 0.23	41.71 ± 0.50	61.90 ± 0.81	51.72 ± 0.16	48.50 ± 0.23	108.42 ± 0.79	86.84 ± 0.43	79.15 ± 0.17
23	swertiajaponin ^t^	37.4	270, 348	461	341b, 313, 297	93.05 ± 1.33	72.63 ± 0.86	104.72 ± 0.67	7.91 ± 0.08	40.19 ± 0.38	115.10 ± 0.45	82.17 ± 0.57	80.45 ± 1.11
24	isovitexin ^s^	40.8	269, 337	431	413, 341, 311b	4.67 ± 0.15	1.87 ± 0.03	1.28 ± 0.11	4.20 ± 0.09	-	-	-	-
25	phenolic acid derivative	40.8	267, 330	545	375b, 195	2.19 ± 0.02	0.98 ± 0.01	1.12 ± 0.02	2.83 ± 0.08	-	-	-	-
26	quercetin 3-*O*-galactoside (hyperoside) ^s^	41.1	260, 353	463	301b, 179	0.86 ± 0.03	-	-	-	-	-	-	-
27	ferulic acid ^s^	41.7	300sh, 325	193	-	-	1.16 ± 0.05	-	-	-	-	-	-
28	apigenin derivative	42.1	269, 333	445	413, 325b, 293, 231	8.53 ± 0.15	5.97 ± 0.11	8.17 ± 0.09	-	-	12.41 ± 0.21	10.69 ± 0.13	9.66 ± 0.07
29	undefined iridoid	44.6	240	527	365b, 295, 179	4.63 ± 0.08	4.27 ± 0.02	1.17 ± 0.05	-	-	-	-	-
30	phenolic acid derivative	45.8	300sh, 322	587	555, 375b, 195	1.73 ± 0.03	-	-	-	-	1.52 ± 0.08	1.27 ± 0.05	1.61 ± 0.05
31	flavonoid derivative with *p*-coumaroyl moiety	46.1	282, 315	747	601, 585b, 485, 357, 227	13.02 ± 0.12	23.19 ± 0.35	14.09 ± 0.17	196.02 ± 0.62	10.51 ± 0.27	8.17 ± 0.15	10.05 ± 0.18	11.76 ± 0.23
32	3,5-*O*-dicaffeoylquinic acid ^c^	47.3	300sh, 324	515	353b, 191, 179	64.93 ± 0.25	58.35 ± 0.11	70.26 ± 0.36	48.30 ± 1.01	41.29 ± 0.18	105.75 ± 0.37	118.90 ± 0.46	73.53 ± 1.12
33	undefined compound	49.3	225	745	583b, 565, 513, 459	1.94 ± 0.03	2.47 ± 0.05	-	31.52 ± 0.17	-	-	-	-
34	4,5-*O*-dicaffeoylquinic acid ^c^	50.9	300sh, 325	515	353b, 179, 173	17.81 ± 1.01	8.07 ± 0.35	9.75 ± 0.62	7.65 ± 0.30	7.18 ± 0.15	11.57 ± 0.06	13.43 ± 0.20	12.12 ± 0.08
35	phenolic acid derivative	54.9	290, 324	631	583, 373, 193	-	-	-	92.85 ± 0.71	-	0.78 ± 0.02	1.79 ± 0.05	0.83 ± 0.01
36	undefined compound	55.6	233	791	630b, 495, 459, 419, 239	1.18 ± 0.04	1.54 ± 0.02	1.38 ± 0.02	-	-	-	-	-
37	undefined compound	57.6	225	633	419b, 239	-	-	-	0.81 ± 0.01	-	-	-	-
38	undefined compound	62.2	227	629	597,419b,239	-	-	-	0.49 ± 0.02	-	-	-	-
39	tiliroside ^s^	63.0	260, 316	593	447,285b	-	-	-	-	-	0.57 ± 0.05	0.61 ± 0.02	0.44 ± 0.02
40	flavonoid derivative with *p*-coumaroyl moiety	69.1	261, 315	635	575,489,349,285b	-	-	-	-	-	20.20 ± 0.19	23.52 ± 0.25	7.92 ± 0.05
41	flavonoid derivative with *p*-coumaroyl moiety	69.5	261, 315	635	575, 489, 349, 285b	-	-	-	-	-	7.52 ± 0.11	8.12 ± 0.09	8.27 ± 0.01

b—base peak (the most abundant ion in recorded spectrum), ^c^—assignment according to Clifford et al. [31], ^s^—comparison with the chemical standard was made, ^f^—M+HCOO ion, sh—shoulder in UV-Vis spectrum, ^t^—tentative assignment based on literature research; “-”—not detected. The contents of flavonoid derivatives were calculated from the curve of luteolin, iridoids from the aucubin curve, phenolic acids and undefined compounds from chlorogenic acid curve. Each value represents mean content of the compound in µg per 1 g of dried extract ± SD (n = 3).

**Table 2:** Scavenging activity of extracts of *C. uralensis* and *C. gigantea* against DPPH^●^ and ABTS^●+^ radicals and their ferric ion-chelating capacity (CHEL).

Extract	DPPH^●^ [mg/mL ± SD]	ABTS^●+^ [mg/mL ± SD]	CHEL [mg/mL ± SD]
CUA	4.42 ± 0.53 ***	0.61 ± 0.12 **	0.49 ± 0.17
CUM	2.86 ± 0.12 ***	0.58 ± 0.16 **	0.25 ± 0.11
CUE	5.55 ± 0.10 ***	0.72 ± 0.11 ***	1.01 ± 0.13 **
CUMf	6.07 ± 0.40 ***	0.45 ± 0.21 *	1.20 ± 0.13 ***
CUEf	6.87 ± 0.72 ***	0.66 ± 0.27 ***	1.07 ± 0.25 ***
CGA	5.56 ± 0.23 ***	0.53 ± 0.16 **	4.04 ± 0.53 ***
CGM	7.28 ± 0.52 ***	0.54 ± 0.21 **	1.32 ± 0.70 ***
CGE	4.90 ± 0.62 ***	0.57 ± 0.33 **	2.95 ± 0.13 ***
AA	0.48 ± 0.30	-	-
Trolox	-	0.09 ± 0.10	-
Na_2_EDTA·2H2O	-	-	0.01 ± 0.01

* *p* < 0.05, ** *p* < 0.01, *** *p* < 0.001 in comparison with the positive control.

**Table 3:** Inhibition of COX-1 and COX-2 activity of the *C. uralensis* and *C. gigantea* extracts.

Extract	COX-1 Inibition [% ± SD]		COX-2 Inhibition [% ± SD]	
	50 µg/mL	100 µg/mL	50 µg/mL	100 µg/mL
CUA	56.99 ± 1.81 ***	90.82 ± 3.43 ***	60.60 ± 2.46 ***	91.12 ± 4.38
CUM	80.07 ± 2.65 **	97.90 ± 2.32 ***	68.31 ± 1.34 ***	94.85 ± 3.29 *
CUE	76.69 ± 1.29	92.54 ± 3.28 ***	71.52 ± 2.38 ***	75.31 ± 2.15 **
CUMf	74.44 ± 3.18	94.64 ± 2.26 ***	55.24 ± 1.10 ***	87.50 ± 2.29
CUEf	46.07 ± 2.25 ***	67.80 ± 2.34	23.52 ± 1.37 ***	35.33 ± 0.93 ***
CGA	32.23 ± 1.28 ***	45.44 ± 1.23 ***	52.28 ± 2.19 ***	55.75 ± 1.26 ***
CGM	17.01 ± 0.79 ***	31.04 ± 1.15 ***	22.83 ± 1.13 ***	43.07 ± 2.25 ***
CGE	50.99 ± 1.35 ***	58.11 ± 3.36 ***	69.00 ± 2.27 ***	81.49 ± 1.28

* *p* < 0.05, ** *p* < 0.01, *** *p* < 0.001 in comparison to the positive control [Indomethacin (5 µM)–72.79 ± 2.08% (COX-1); 87.31 ± 3.04% (COX-2)].

**Table 4:** Zones of bacterial growth inhibition as an antibacterial *Cephalaria uralensis* and *C. gigantea* extracts activity.

Bacterial Strains	Zones of Bacterial Growth Inhibition [mm]							
	CUA	CUM	CUE	CUMf	CUEf	CGA	CGM	CGE
*S. aureus* ATCC 25923	13	17	12	13.5	10	8	7	8
*S. epidermidis* ATCC 12228	12.5	19	14.5	10.5	10	10	8	10
*P. acnes PCM 2400*	18	23	15	13	11	6	4	8
*P. acnes PCM 2334*	17.5	21.5	16	13	11.5	8	7	10

**Table 5:** Minimum inhibitory concentration (MIC [μg/mL]) and MBC/MIC ratio as an antibacterial activity of the tested *C. uralensis* and *C. gigantea* extracts.

Bacterial Strains	CUA		CUM		CUE		CUMf		CUEf		CGA		CGM		CGE	
	MIC	MBC/MIC	MIC	MBC/MIC	MIC	MBC/MIC	MIC	MBC/MIC	MIC	MBC/MIC	MIC	MBC/MIC	MIC	MBC/MIC	MIC	MBC/MIC
*S. aureus* ATCC 25923	50	8	25	8	100	8	50	8	200	8	>200	-	>200	-	>200	-
*S. epidermidis* ATCC 12228	100	8	25	8	50	8	100	8	200	8	>200	-	>200	-	>200	-
*P. acnes PCM 2400*	25	8	12.5	2	50	8	50	8	100	8	>200	-	>200	-	>200	-
*P. acnes PCM 2334*	25	8	12.5	4	50	8	50	8	100	8	>200	-	>200	-	200	-

**Table 6:** Interactions between *C. uralensis* extracts and selected antibiotics against acne bacteria.

Bacterial Strains	FICI^a^ Index of Different Combinations of Antibiotics and *C. uralensis* Extracts											
	CUA				CUM				CUE			
	Cefepime	Ceftriaxone	Ciprofloxacin	Sparfloxacin	Cefepime	Ceftriaxone	Ciprofloxacin	Sparfloxacin	Cefepime	Ceftriaxone	Ciprofloxacin	Sparfloxacin
*S. aureus*	2 ^d^	1.5 ^d^	1.06 ^d^	1.5 ^d^	2 ^d^	1.06 ^d^	1.5 ^d^	1.5 ^d^	1.5 ^d^	2 ^d^	2 ^d^	2 ^d^
*S. epidermidis*	1.06 ^d^	1.5 ^d^	1.06 ^d^	1.5 ^d^	1.06 ^d^	1.06 ^d^	1.5 ^d^	1.06 ^d^	1.5 ^d^	1.5 ^d^	1.5 ^d^	2 ^d^
*P. acnes PCM 2400*	1.06 ^d^	2 ^d^	2 ^d^	1.5 ^d^	0.5 ^b^	0.5 ^b^	1.0 ^d^	1.06 ^d^	1.5 ^d^	1.5 ^d^	1.06 ^d^	1.5 ^d^
*P. acnes PCM 2334*	1.06 ^d^	1.06 ^d^	1.5 ^d^	1.06 ^d^	0.5 ^b^	0.375 ^b^	0.562 ^c^	1.06 ^d^	1.06 ^d^	1.5 ^d^	1.06 ^d^	1.06 ^d^

^a^ Fractional inhibitory concentration index; ^b^ Total synergism; ^c^ Partial synergism; ^d^ No effect.

**Table 7:** The half-maximal cytotoxic concentrations (CC_50_) and therapeutic indexes (TI) of the most active extracts (CUA, CUM, CUE, CUMf, CUEf).

Extract	CC_50_ [μg/mL]	TI $\frac{CC_{50}}{MIC}$			
		*S. aureus* ATCC 25923	*S. epidermidis* ATCC 12228	*P. acnes* PCM 2400	*P. acnes* PCM 2334
CUA	191.60 ± 3.25	3.83	1.92	7.66	7.66
CUM	61.27 ± 2.74	2.45	2.45	4.90	4.90
CUE	> 1000	**> 10**	**> 20**	**> 20**	**> 20**
CUMf	72.09 ± 2.16	1.44	0.72	1.44	1.44
CUEf	> 1000	> 5	> 5	**> 10**	**>10**

The CC_50_ values were determined towards normal human skin fibroblasts (BJ cell line, ATTC CRL-2522^™^) after 24-h incubation. The TI values were calculated as the ratio between CC_50_ and MIC values.

## References

[1] https://www.reportlinker.com/p05251482.

[2] African O., Kurutas E.B., Sasmaz S. (2005). Oxidative stress in patients with acne vulgaris. Mediat. Inflamm..

[3] Shaw L., Kennedy C. (2007). The treatment of acne. Paediatr. Child. Health..

[4] Dessinioti C., Katsambas A. (2013). Seborrheic dermatitis: Etiology, risk factors, and treatments: Facts and controversies. Clin. Dermatol..

[5] Foti C., Romita P., Borghi A., Angelini G., Bonamonte D., Corazza M. (2015). Contact dermatitis to topical acne drugs: A review of the literature. Dermatol. Ther..

[6] Humphrey S. (2012). Antibiotic resistance in acne treatment. Skin Ther. Lett..

[7] Zayyad H., Eliakim-Raz N., Leibovici L., Paul M. (2017). Revival of old antibiotics: Needs, the state of evidence and expectations. Int. J. Antimicrob. Agents.

[8] Kong D.-X., Li X.-J., Zhang H.-Y. (2009). Where is the hope for drug discovery? Let history tell the future. Drug Discov. Today.

[9] Patwardhan B. (2005). Traditional Medicine: Modern Approach for Affordable Global Health.

[10] Patwardhan B., Mashelkar R.A. (2009). Traditional medicine—inspired approaches to drug discovery: Can Ayurveda show the way forward?. Drug Discov. Today.

[11] Harvey A.L., Edrada-Ebel R.A., Quinn R.J. (2015). The re-emergence of natural products for drug discovery in the genomic era. Nat. Rev. Drug Discov..

[12] Baydoun S., Lamis C., Dalleh H., Nelly A. (2015). Ethnopharmacological survey of medicinal plants used in traditional medicine by the communities of Mount Hermon, Lebanon. J. Ethnopharmacol..

[13] Dalar A., Mukemre M., Unal M., Ozgokce F. (2018). Traditional medicinal plants of Ağrı Province, Turkey. J. Ethopharmacol..

[14] Nasri H., Bahmani M., Shahinfard N., Nafchi A.M., Saberianpour S., Kopaei M.R. (2015). Medicinal plants for the treatment of acne vulgaris: A review of recent evidences. Jundishapur J. Microbiol..

[15] Tabassum N., Hamdani M. (2014). Plants used to treat skin diseases. Pharmacogn. Rev..

[16] Pieroni A., Quave C.L., Villanelli M.L., Mangino P., Sabbatini G., Santini L., Boccetti T., Profili M., Ciccioli T., Rampa L.G. (2004). Ethnopharmacognostic survey on the natural ingredients used in folk cosmetics, cosmeceuticals and remedies for healing skin diseases in the inland Marches, Central-Eastern Italy. J. Ethnopharmacol..

[17] Sarıkahya N.B., Kırmızıgül S. (2012). Antimicrobially active hederagenin glycosides from *Cephalaria elmaliensis*. Planta Med..

[18] Sumer G., Sarikahya N.B., Kirmizigul S. (2017). Phytochemical and biological investigations on *Cephalaria anatolica*. Rec. Nat. Prod..

[19] Kayce P., Kırmızıgül S. (2010). Chemical constituents of two endemic *Cephalaria* species. Rec. Nat. Prod..

[20] Alankuş-Çalişkan Ö., Anil H. (1995). A bidesmodic triterpene saponin from *Cephalaria transsyvanica*. Phytochemistry.

[21] Celenk V.U., Sarikahya N.B., Kirmizigul S. (2020). Isolation and structural studies on saponins from three *Cephalaria* species from Anatolia. Chem. Nat. Compd..

[22] Kırmızıgül S., Anıl H., Uçar F., Akdemir K. (1996). Antimicrobial and antifungal activities of three new triterpenoid glycosides. Phytoter. Res..

[23] Top H., Sarıkahya N.B., Nalbantsoy A., Kırmızıgül S. (2017). Immunomodulatory, hemolytic properties and cytotoxic activity potent of triterpenoid saponins from *Cephalaria balansae*. Phytochemistry.

[24] Mustafayeva K., Elias R., Balansard G., Suleimanov T., Mayu-Lede V., Kerimov Y. (2008). Iridoid glycosides from *Cephalaria kotschyi* roots. Chem. Nat. Compd..

[25] Aliev A.M., Movsumov I.S., Bagirov E.K. (1975). Alkaloids from certain *Cephalaria* species. Khim. Prir. Soedin..

[26] Movsumov I.S., Garaev E.A., Isaev M.I. (2009). Flavonoids from *Cephalaria grossheimii*. Chem. Nat. Compd..

[27] Kayce P., Sarikahya B.N., Pekmez M., Arda N., Kırmızıgül S. (2017). The structure and cytotoxic activity of a new saponin: Cephoside A from *Cephalaria elazigensis* var. purpurea. Turk. J. Chem..

[28] Sarıkahya N.B., Kırmızıgül S. (2010). Antimicrobial triterpenoid glycosides from *Cephalaria scoparia*. J. Nat. Prod..

[29] Kırmızıgül S., Anıl H., Rose M.E. (1996). Triterpenoid saponins from *Cephalaria transsylvanica*. J. Nat. Prod..

[30] Hamdan I.I., Afifi F.U. (2004). Studies on the in vitro and in vivo hypoglycemic activities of some medicinal plants used in treatment of diabetes in Jordanian traditional medicine. J. Ethopharmacol..

[31] (2011). Polish Pharmacopoeia IX, PTFarm.

[32] Szewczyk K., Bogucka-Kocka A., Vorobets N., Grzywa-Celińska A., Granica S. (2020). Phenolic composition of the leaves of *Pyrola rotundifolia* L. and their antioxidant and cytotoxic activity. Molecules.

[33] Guo J.T., Lee H.L., Chiang S.H., Lin H.I., Chang C.Y. (2001). Antioxidant properties of the extracts from different parts of broccoli in Taiwan. J. Food Drug Anal..

[34] Murray P.R., Baron E.J., Pfaller M.A., Tenover F.C., Yolke R.H. (1995). Manual of Clinical Microbiology.

[35] Clinical Laboratory Standards Institute (2008). Performance Standards for Antimicrobial Susceptibility Testing. Eighteenth International Supplement.

[36] Fadli M., Saad A., Sayadi S., Chevalier J., Mezrioui N.E., Pages J.M., Hassani L. (2012). Antibacterial activity of *Thymus maroccanus* and *Thymus broussonetii* essential oils against nosocomial infection—bacteria and their synergistic potential with antibiotics. Phytomedicine.

[37] Miazga-Karska M., Szewczyk K., Klimek K., Ginalska G. (2017). In vitro activity of peptide fractions from *Impatiens glandulifera* against caries causing bacteria. Acta Pol. Pharm..

[38] Pitucha M., Woś M., Miazga-Karska M., Klimek K., Mirosław B., Pachuta-Stec A., Gładysz A., Ginalska G. (2016). Synthesis, antibacterial and antiproliferative potential of some new 1-pyridinecarbonyl-4-substituted thiosemicarbazide derivatives. Med. Chem. Res..

[39] Clifford M.N., Johnston K.L., Knight S., Kuhnert N. (2003). Hierarchical scheme for LC-MS*^n^* identification of chlorogenic acids. J. Agric. Food Chem..

[40] Clifford M.N. Some Notes on the Chlorogenic Acids. 3. LC and LC–MS. Version 3. https://www.researchgate.net/publication/312590947_Some_Notes_on_the_Chlorogenic_Acids_3_LC_and_LC-MS_Version_3_January_2017.

[41] Clifford M.N., Knight S., Kuhnert N. (2005). Discriminating between the six isomers of dicaffeoylquinic acid by LC-MS(n). J. Agric. Food Chem..

[42] Zemtsova G.N., Bandyukova V.A. (1970). A chemical study of *Cephalaria gigantea*. Chem. Nat. Compd..

[43] Pereira C.A., Yariwake J.H., McCullagh M. (2005). Distinction of the C-glycosylflavone isomer pairs orientin/isoorientin and vitexin/isovitexin using HPLC-MS exact mass measurement and in-source CID. Phytochem. Anal..

[44] Sarıkahya N.B., Goren A.C., Kırmızıgül S. (2019). Simultaneous determination of several flavonoids and phenolic compounds in nineteen different *Cephalaria* species by HPLC-MS/MS. J. Pharm. Biomed. Anal..

[45] Grochowski D.M., Locatelli M., Granica S., Cacciagrano F., Tomczyk M. (2018). A review on the dietary flavonoid tiliroside. Compr. Rev. Food Sci. Food Saf..

[46] Mustafayeva K., Mahiou-Leddet V., Suleymanov T., Kerimov Y., Ollivier R., Elias R. (2011). Chemical constituents from the roots of *Cephalaria kotschyi*. Chem. Nat. Compd..

[47] Rønsted N., Göbel E., Franzyk H., Jensen S.R., Olsen C.E. (2000). Chemotaxonomy of *Plantago*. Iridoid glucosides and caffeoyl phenylethanoid glycosides. Phytochemistry.

[48] Sarıkahya N.B., Pekmez M., Arda N., Kayce P., Yavaşoğlu N.Ü.K., Kırmızıgül S. (2011). Isolation and characterization of biologically active glycosides from endemic *Cephalaria* species in Anatolia. Phytochem. Lett..

[49] Gaweł-Bęben K., Strzępek-Gomółka M., Czop M., Sakipova Z., Głowniak K., Kukula-Koch W. (2020). *Achillea millefolium* L. and *Achillea biebersteinii* Afan. hydroglycolic extracts–bioactive ingredients for cosmetic use. Molecules.

[50] Magnani C., Isaac V.L.B., Correa M.A., Salgado H.R.N. (2014). Caffeic acid: A review of its potential use in medications and cosmetic. Anal. Methods.

[51] Sarici G., Cinar S., Armutcu F., Altınyazar C., Koca R., Tekin N.S. (2010). Oxidative stress in acne vulgaris. J. Eur. Acad. Dermatol. Venereol..

[52] Azab A. (2018). Total phenolic content, antioxidant capacity and antifungal activity of extracts of *Carthamus tenuis* and *Cephalaria joppensis*. Eur. Chem. Bull..

[53] Campos J., Schmeda-Hirschmann G., Leiva E., Guzmán L., Orrego R., Fernández P., González M., Radojkovic C., Zuñiga F.A., Lamperti L. (2014). Lemon grass (*Cymbopogon citratus* (D.C.) Stapf) polyphenols protect human umbilical vein endothelial cell (HUVECs) from oxidative damage induced by high glucose, hydrogen peroxide and oxidized low-density lipoprotein. Food Chem..

[54] Thiboutot D.M. (1997). Acne: An overview of clinical research findings. Dermatol. Clin..

[55] Gautam R., Srivastava A., Jachak S.M., Saklani A. (2010). Anti-inflammatory, cyclooxygenase (COX)-2, COX-1 inhibitory and antioxidant effects of *Dysophylla stellate* Benth. Fitoterapia.

[56] Eldeen I.M.S., Van Staden J. (2008). Cyclooxygenase inhibition and antimycobacterial effects of extracts from Sudanese medicinal plants. S. Afr. J. Bot..

[57] Lu J., Fu X., Liu T., Zheng Y., Chen J., Luo F. (2018). Phenolic composition, antioxidant, antibacterial and anti-inflammatory activities of leaf and stem extracts from *Cryptotaenia japonica* Hassk. Ind. Crop. Prod..

[58] Almeida I.F., Valentão P., Andrade P.B., Seabra R.M., Pereira T.M., Amaral M.H., Costa P.C., Bahia M.F. (2008). In vivo skin irritation potential of a *Castanea sativa* (chestnut) leaf extract, a putative natural antioxidant for topical application. Basic. Clin. Pharmacol..

[59] Jibry N., Murdan S. (2004). In vivo investigation, in mice and in man, into the irritation potential of novel amphiphilogels being studied as transdermal drug carriers. Eur. J. Pharm. Biopharm..

